# Combination of neutrophil to lymphocyte ratio, platelet to lymphocyte ratio with plasma D-dimer level to improve the diagnosis of deep venous thrombosis (DVT) following ankle fracture

**DOI:** 10.1186/s13018-023-03840-3

**Published:** 2023-05-16

**Authors:** Zhida Gao, Kuo Zhao, Lin Jin, Xiaodong Lian, Zhiang Zhang, Lijie Ma, Zhiyong Hou

**Affiliations:** 1 Department of Orthopaedic Surgery, Third Hospital of Hebei Medical University, Shijiazhuang, 050051 Hebei People’s Republic of China; 2 Department of Orthopaedic Surgery, Shijiazhuang People’s Hospital, Shijiazhuang, 050051 Hebei People’s Republic of China; 3 Key Laboratory of Biomechanics of Hebei Province, Shijiazhuang, 050051 Hebei People’s Republic of China; 4 Orthopaedic Research Institution of Hebei Province, Shijiazhuang, 050051 Hebei People’s Republic of China; 5 NHC Key Laboratory of Intelligent Orthopaedic Equipment, The Third Hospital of Hebei Medical University, Shijiazhuang, People’s Republic of China; 6 Department of Orthopaedic Trauma Center, The 3rd Hospital of Hebei Medical University, No 139 Ziqiang Road, Shijiazhuang, 050051 Hebei People’s Republic of China

**Keywords:** Ankle fracture, Lymphocyte ratio/platelet to lymphocyte ratio, Combination diagnostic model, Improved diagnostic performance

## Abstract

**Purpose:**

To investigate the relationship between neutrophil to lymphocyte ratio (NLR)/platelet to lymphocyte ratio (PLR) with deep venous thrombosis (DVT) following ankle fracture and the diagnostic ability of combination model.

**Method:**

This retrospective study included patients with a diagnosis of ankle fracture who had undergone preoperative Duplex ultrasound (DUS) examination for detecting the possible deep venous thrombosis (DVT). The variables of interest, the calculated NLR and PLR and others (demographics, injury, lifestyles and comorbidities) were extracted from the medical records. Two independent multivariate logistics regression models were used to detect the relationship between NLR or PLR and DVT. If any, combination diagnostic model was constructed and its diagnostic ability was evaluated.

**Results:**

There were 1103 patients included, and 92 (8.3%) were found to have preoperative DVT. The NLR and PLR, which had respective optimal cut-off point of 4 and 200, were significantly different between patients with and without DVT either in continuous or categorical variable. After adjustment for covariates, both NLR and PLR were identified as independent risk factors associated with DVT, with odd ratio of 2.16 and 2.84, respectively. The combination diagnostic model, including NLR, PLR and D-dimer, demonstrated to significantly improved the diagnostic performance than any one alone or combined (all *P* < 0.05), and the area under the curve was 0.729 (95% CI 0.701–0.755).

**Conclusion:**

We concluded the relatively low incidence rate of preoperative DVT after ankle fracture, and both NLR and PLR were independently associated with DVT. The combination diagnostic model can be considered as a useful auxiliary tool for identifying high-risk patients for DUS examination.

## Introduction

Deep venous thrombosis (DVT) is a prevalent and severe complication in orthopedic trauma, especially in lower extremity fractures. Ankle fracture is among the most common injuries and the preoperative incidence of DVT is greatly varying from 0.28 to 13.7%, primarily attributable to the differences in study design, setting, patient characteristics, screening methods and the prevention strategies [1–4]. DVT was associated with significantly increased risk of adverse events, including pulmonary emboli, atherothrombosis and cardiovascular complications [5, 6]. To date, DVT prophylaxis is not routinely administered in ankle fracture surgery in most institutions, when balancing the risks and benefits. In contrast, identifying individuals at high-risk and establishing tailored early detection and prevention strategies has been consistently the most desirable approach.

The plasma D-dimer test is generally used in various settings as a primary screening test for detection of DVT, pulmonary embolism (PE) or both, due to its ease, high sensitivity and low-cost of operation. However, the low specificity (even < 20% even in elderly patients) will produce a disproportionate number of false positives, and thus recur additional medical resources consumption [7]. During the past decade, efforts to identify a variety of risk factors associated with DVT have been made, and some important factors have been well established such as older age, male gender, higher fracture severity, history of VTE, immobility of injured extremity and et al. [1, 2, 8–10]. Recently, emerging evidences have shown that DVT was associated with inflammatory/immune response to fracture, surgical trauma or systemic chronic conditions in different medical settings [11–13]. Neutrophil to lymphocyte ratio (NLR) and platelet to lymphocyte ratio (PLR) are the typical representatives [14, 15], but their relation to DVT is not always consistent. Considering that these two indexes are readily accessible, low-cost and without requiring additional laboratory test, it is very essential to elucidate their relationship with DVT, and if any, to investigate the potential of combination diagnosis by using NLR/PLR and the D-dimer level. This study therefore, aimed to first, determine whether NLR or PLR was associated with preoperative presence of DVT, and second, if any, to evaluate the diagnostic ability of NLR and/or PLR with plasma D-dimer level.

## Materials and methods

### Inclusion and exclusion criteria

This study was approved by the ethics committees of the Third Hospital of Hebei Medical University and was performed in accordance with the Helsinki Declaration. Adult patients who had an ankle fracture surgically treated and had a complete preoperative duplex ultrasound (DUS) screening examination between January 2017 and December 2021 in our institution were included. The exclusions criteria were old fracture (> 21 days after fracture), pathological fracture, open fracture, multiple fractures, polytrauma, abnormal lower limb muscle strength, history of VTE events, thrombophilia or hematological disorders, anticoagulants or glucocorticoids use within 3 months of fracture, anticoagulant medication administered before DUS screening or absence of preoperative DUS examination. According to the Robinov group’s criteria, the diagnosis of DVT was made by two ultrasound physicians [16].

### Measurement of biological indicators

**Fig. 1:**
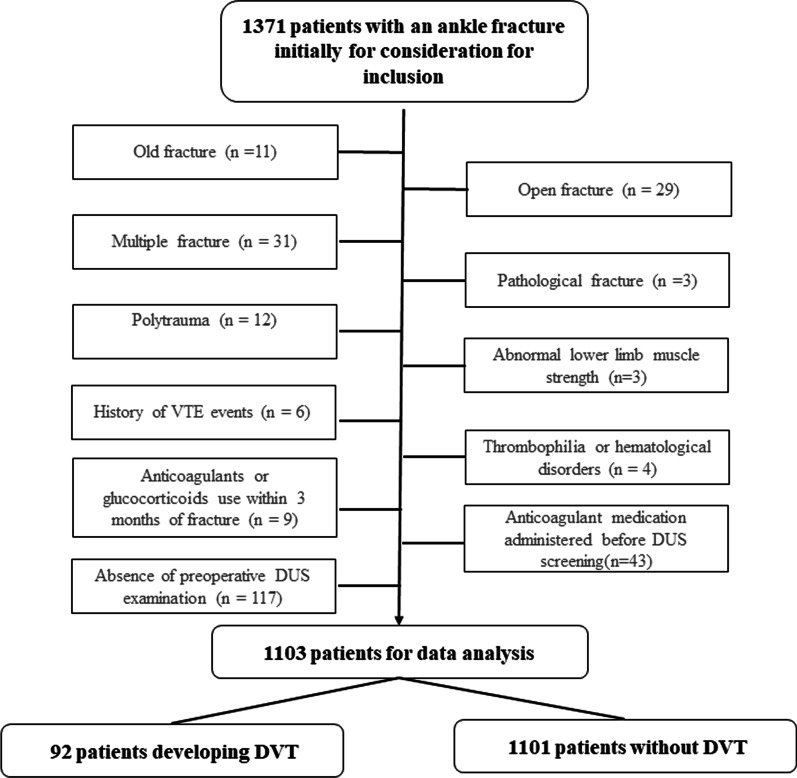
The flowchart of this study

Routine blood test was performed immediately after admission and repeated before operation in accordance with the Instruction Manual, by use of a hematology analyzer (UniCel DxH 800; Beckman Coulter, Brea, CA, USA) an automated coagulation analyzer (ACL 700, Beckman Coulter, Brea, CA, USA), respectively. NLR was calculated by dividing the neutrophil count by the lymphocyte count, and PLR was calculated by dividing the platelet count by the lymphocyte count (Fig. 1).

### Data collection

Data were collected by inquiring the electronic medical record for the index hospitalization. These included age at the time of surgery, gender, time from injury to DUS examination, body mass index (BMI, calculated by dividing the body weight in kilogram by the height in meter), fracture location (uni-, bi- or tri-malleolar), presence or absence of dislocation/subluxation, injury mechanism (high- or low-energy trauma), smoking status (yes or no), alcohol drinking (yes or no), comorbidities (hypertension, diabetes, cerebrovascular disease, heart disease, living disease), American Society of Anesthesiologists (ASA) classification, lymphocyte count, neutrophil count, platelet count and plasma D-dimer concentration.

### Statistical analysis

For continuous variables, Shapiro–Wilk test was used to explore their normally distributed status, based on which, continuous variables were expressed as mean ± standard deviation (SD) for normally distributed data or median and interquartile range (IQR) for the skewed. Student-*t* test or Mann–Whitney *U* test was performed to compare the between-group difference, as appropriate. Categorical variables were expressed as number and its percentage, and Chi-square or Fisher’s exact test was used to compare the between-group difference, as appropriate.

First, receiver operator characteristic (ROC) analysis was performed to determine the optimal cut-off value for NLR, PLR and plasma D-dimer, when Youden index (= sensitivity + specificity-1) was maximum. Based on the cut-off value, these three indexes were dichotomized as low versus high, respectively. The area under the ROC curve (AUC) was to quantify the diagnostic ability, which ranged from 0 to 100%, with higher representing better ability. To investigate whether NLR in dichotomous variable was independently associated with preoperative DVT, a multivariate logistics regression models were constructed, when adjusting for all the covariates (not including PLR) using the “enter” method, namely the “total adjusted model”, did as the same for PLR. The association magnitude was indicated by odd ratio (OR) with 95% confidence interval (95% CI). The goodness-of-fit of the model was evaluated by Hosmer–Lemeshow (H–L) test, with *P* > 0.05 suggestive of an acceptable result. The above analyses were performed using SPSS 23.0 (IBM, Armonk, New York, USA).

If the association of NLR and/or PLR with preoperative DVT demonstrated to be significant, another multivariate logistic regression model adjusted for NLR and/or PLR and D-dimer was constructed, forming the combination diagnostic model and the C-statistic (equivalent to the AUC) was used to evaluate the diagnostic performances of this combination diagnostic model. Comparisons of diagnostic performances between D-dimer alone, combination with PLR and/or NLR in diagnosing preoperative DVT were performed by using the MedCalc software (MedCalc 19.2.1; MedCalc, Mariakerke, Belgium).

The *P* < 0.05 was set as significance level for all the analyses.

## Results

There were 1103 patients included in this study, including 657 males and 446 females, with an average age of 42.8 ± 14.2 years (range, 18–84). Among them, 92 were found to have preoperative DVT detected by DUS, suggesting an incidence rate of 8.3% (95% CI 6.7–10.0%). A total of 142 thrombi were found, indicating an average of 1.54 for each patient with DVTs; most of thrombi (92.3%, 131) were located distal to the popliteal veins, and over 80% (81.7%, 116/142) at the injured limb.

There were significantly differences between patients with and without DVT in terms of age (48.6 ± 14.7 vs 42.3 ± 14.0), male gender (70.7% vs 58.6%), prevalence of hypertension (19.6% vs 12.3%), injury mechanism (65.5% vs 35.5%), lymphocyte count (1.3 ± 0.6 vs 1.6 ± 0.7), NLR (5.7 ± 3.1 vs 4.7 ± 3.2), PLR (225.7 ± 125.4 vs 167.1 ± 80.9), and D-dimer concentration (2.6 ± 5.8 vs 0.8 ± 1.5), with all *P* values less than 0.05 (Table 1).

**Table 1:** Univariate analysis of variables between DVT and non-DVT group

Variables	Number (%) of patients without DVT (*n* = 1011)	Number (%) of patients with DVT (*n* = 92)	*P*
Age (years)	42.3 ± 14.0	48.6 ± 14.7	< 0.001
Sex (male)	592 (58.6)	65 (70.7)	0.024
BMI (kg/m^2^)	25.6 ± 3.9	26.0 ± 3.0	0.667
< 28.0	877 (86.7)	79 (85.9)	0.813
≥ 28.0	134 (13.3)	13 (14.1)	
Fracture location			
Unimalleolar	427 (42.2)	44 (47.8)	0.583
Bimalleolar	244 (24.1)	20 (21.7)	
Trimalleolar	340 (33.6)	28 (30.4)	
Dislocation/subluxation	193 (19.1)	23 (25.0)	0.171
Hypertension	124 (12.3)	18 (19.6)	0.045
Diabetes mellitus	146 (14.4)	20 (21.7)	0.061
Cerebrovascular disease	9 (0.9)	2 (2.2)	0.235
Heart disease	36(3.6)	3(3.3)	0.881
Liver disease	22 (2.2)	4 (4.3)	0.189
Smoking	250 (24.7)	25 (27.2)	0.604
Alcohol drinking	280 (27.7)	32 (34.8)	0.148
Injury mechanism			0.001
Low-energy	652 (64.5)	43 (46.7)	
High-energy	359 (35.5)	49 (53.3)	
ASA classification			0.122
I	180 (17.8)	13 (14.1)	
II	715 (70.7)	62 (67.4)	
III–IV	116 (11.5)	17 (18.5)	
Lymphocyte	1.6 ± 0.7	1.3 ± 0.6	< 0.001
Neutrophile	6.4 ± 2.7	6.4 ± 2.1	0.982
Platelet	239.2 ± 80.0	259.8 ± 101.1	0.060
NLR	4.7 ± 3.2	5.7 ± 3.1	0.004
≥ 4	492 (48.7)	64 (69.6)	< 0.001
PLR	167.1 ± 80.9	225.7 ± 125.4	< 0.001
≥ 200	253 (25.0)	47 (51.1)	< 0.001
D-dimer	0.8 ± 1.5	2.6 ± 5.8	0.004
≥ 0.80	259 (25.6)	54 (58.7)	< 0.001

*DVT*—deep venous thrombosis, *BMI*—body mass index, *ASA*—American Society of Anesthesiologists, *NLR*—neutrophil to lymphocyte ratio, *PLR*—platelet to lymphocyte ratio

**Fig. 2:**
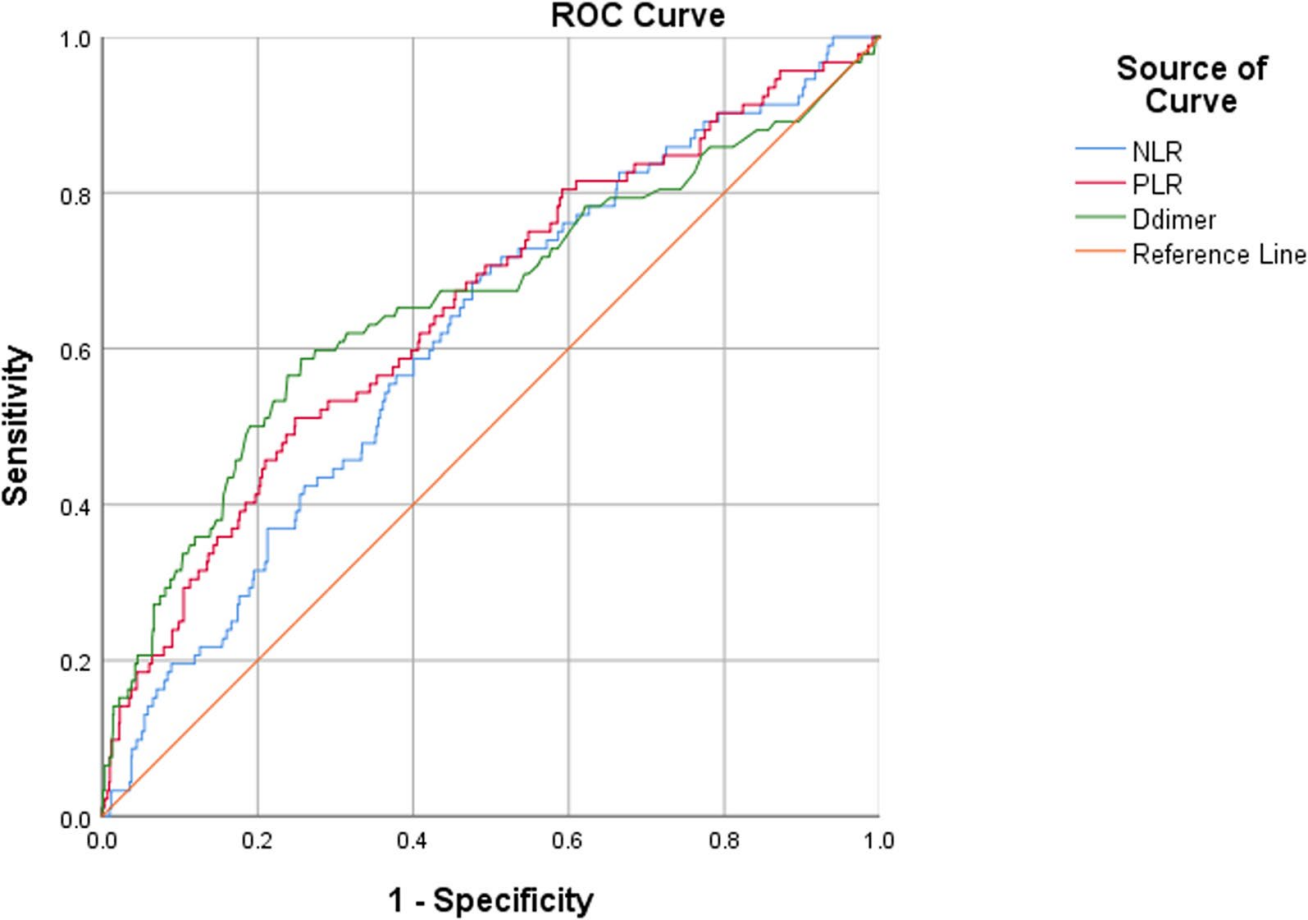
The ROC curve and analysis for NLR, PLR, and D-dimer, respectively. The optimal cut-off point for NLR, PLR, and D-dimer was 4, 200 and 0.8 mg/L, with respective AUC being 0.616 (95% CI 0.557–0.674; *P* < 0.001), 0.653 (95% CI 0.591–0.714; *P* < 0.001), and 0.660 (95% CI 0.631–0.688; *P* < 0.001)

**Fig. 3:**
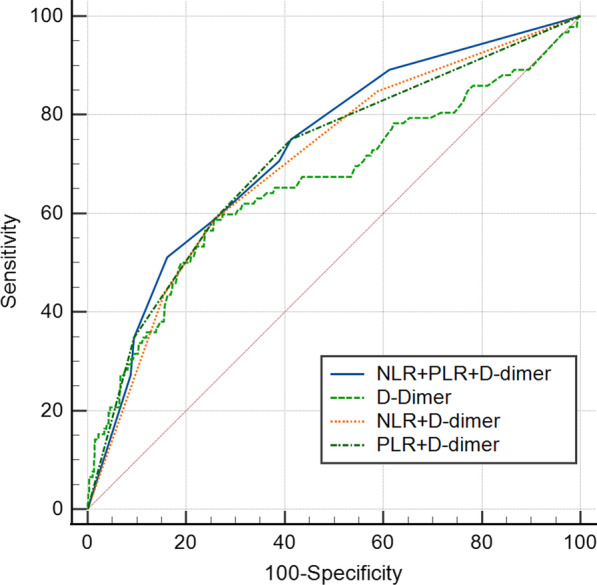
Depicted the ROC curve and analysis for D-dimer, NLR + D-dimer, PLR + D-dimer, and NLR + PLR + D-dimer, with the latter one having the significantly higher diagnostic performance

The NLR was 4.77 ± 3.19 (range 0.35–34.36), with 5.7 ± 3.1 in DVT group and 4.7 ± 3.2 in non-DVT group, and the difference was significant (*P* = 0.004). The optimal cut-off point for NLR was 4 (AUC, 0.616; 95% CI 0.557–0.674; *P* < 0.001) and the proportion of NLR ≥ 4 was 69.6% (64/92) in patients with DVT and 48.7% (492/1011) in those without, with a significant difference (*P* < 0.001) (Fig. 2). The sensitivity, specificity, positive predictive value (PPV), and negative predictive value (NPV) for NLR was 69.6%, 51.3%, 11.5%, and 94.9%. The total adjusted logistic regression model showed NLR ≥ 4 was significantly associated with the increased risk of DVT, with OR value of 2.16 (95% CI 1.34–3.49; *P* = 0.002) (Table 2). The PLR was 172.00 ± 86.97 (range, 13.0–660.0), with 225.7 ± 125.4 in DVT group and 167.1 ± 80.9 in non-DVT group, and the difference was significant (*P* < 0.001). The optimal cut-off point for PLR was 200 (AUC, 0.653; 95% CI 0.591–0.714; *P* < 0.001) and the proportion of PLR ≥ 200 was 51.1% (47/92) in patients with DVT and 25.0% (253/1011) in those without, with a significant difference (*P* < 0.001) (Fig. 2). Also, the total adjusted logistic regression model showed the significant relation between PLR ≥ 200 and DVT, with OR value of 2.84 (95% CI 1.80–4.47; *P* < 0.001) (Table 3). The sensitivity, specificity, PPV, and NPV for PLR was 51.1%, 75.0%, 15.7%, and 94.4%. D-dimer level, which was determined to be with an optimal cut-off point of 0.80 (AUC, 0.660; 95% CI 0.631–0.688; *P* < 0.001), demonstrated to be significantly associated with DVT in both multivariate logistic regression models for NLR and PLR (Table 2s and 3). Figure 3 depicts the AUC, which represented the diagnostic performance in DVT, for four indexes, D-dimer alone (0.660; 95% CI 0.631–0.688), NLR + D-dimer (0.704; 95% CI 0.676–0.731), PLR + D-dimer (0.711; 95% CI 0.683–0.737), and NLR + PLR + D-dimer (0.729; 95% CI 0.701–0.755). The results showed the combination model with D-dimer, NLR, and PLR included exhibited the significantly better diagnostic performance, compared to any other one, single or combined (Table 4). Particularly, compared to the use of D-dimer alone, the combination model showed the significantly larger AUC, with an absolute difference of 0.069 (*P* = 0.003). The sensitivity, specificity, PPV, and NPV for D-dimer was 58.7%, 74.4%, 17.2%, and 95.2%.

**Table 2:** Relationship between NLR and preoperative DVT investigated by total adjusted multivariate logistic regression model

Variables	OR and 95% CI	*P*
NLR	2.16 (1.34–3.49)	0.002
Age	1.04 (1.02–1.05)	< 0.001
Gender (male vs female)	1.95 (1.08–3.53)	0.027
Obesity (BMI ≥ 28.0 kg/m^2^)	1.22 (0.61–2.41)	0.573
Smoking	0.78 (0.42–1.43)	0.414
Alcohol drinking	1.32 (0.74–2.35)	0.350
Time from fracture to DUS examination	1.16 (1.10–1.23)	< 0.001
Fracture type		
Unimalleolar	Reference	
Bimalleolar	0.82 (0.44–1.50)	0.508
Trimalleolar	0.80 (0.45–1.41)	0.432
Dislocation/subluxation	1.27 (0.72–2.22)	0.410
Mechanism (high- versus low-energy)	1.45 (0.88–2.37)	0.144
Hypertension	1.33 (0.70–2.53)	0.378
Diabetes mellitus	1.46 (0.80–2.69)	0.220
Cerebrovascular disease	0.81 (0.15–4.50)	0.811
Heart disease	1.32 (0.43–8.91)	0.090
Liver disease	2.16 (0.66–7.09)	0.205
D-dimer (> 0.80 mg/L)	2.90 (1.77–4.74)	< 0.001

*NLR*—neutrophil to lymphocyte rate, *BMI*—body mass index, *OR*—odd ratio, *CI*—confidence interval

**Table 3:** Relationship between PLR and preoperative DVT investigated by total adjusted multivariate logistic regression model

Variables	OR and 95% CI	*P*
PLR	2.84 (1.80–4.47)	< 0.001
Age	1.04 (1.02–1.05)	< 0.001
Gender (male vs female)	2.12 (1.21–3.76)	0.027
Obesity (BMI ≥ 28.0 kg/m^2^)	1.23 (0.62–2.45)	0.558
Smoking	0.76 (0.42–1.41)	0.387
Alcohol drinking	1.30 (0.73–2.32)	0.380
Time from fracture to DUS examination	1.15 (1.08–1.22)	< 0.001
Fracture type		
Unimalleolar	Reference	
Bimalleolar	0.83 (0.45–1.53)	0.551
Trimalleolar	0.83 (0.47–1.49)	0.538
Dislocation/subluxation	1.33 (0.76–2.33)	0.316
Mechanism (high- versus low-energy)	1.38 (0.84–2.26)	0.202
Hypertension	1.37 (0.72–2.60)	0.342
Diabetes mellitus	1.43 (0.78–2.63)	0.247
Cerebrovascular disease	0.87 (0.16–4.84)	0.872
Heart disease	1.29 (0.41–8.84)	0.088
Liver disease	2.01 (0.61–6.59)	0.251
D-dimer (> 0.80 mg/L)	2.91 (1.79–4.76)	< 0.001

*PLR*—platelet to lymphocyte rate, *BMI*—body mass index, *OR*—odd ratio, *CI*—confidence interval

**Table 4:** Comparison of diagnostic performance by AUC among different markers or combined

Pairwise comparisons	Absolute difference in AUC	Z statistic	*P*
D-dimer versus D-dimer + NLR	0.044	1.959	0.050
D-dimer versus D-dimer + PLR	0.051	2.285	0.022
D-dimer versus D-dimer + NLR + PLR	0.069	2.938	0.003
D-dimer + NLR versus D-dimer + NLR + PLR	0.024	2.235	0.025
D-dimer + PLR versus D-dimer + NLR + PLR	0.018	2.013	0.044
D-dimer + NLR versus D-dimer + PLR	0.007	0.378	0.705

*PLR*—platelet to lymphocyte rate, *NLR*—neutrophil to lymphocyte rate, *AUC*—area under the curve

## Discussion

Given the clinical importance of DVT in short-and long-term prognosis, there has been increasing number of studies focusing on identification of the acquired or inherited risk factors, helping to adopt the optimal preventive strategies in high-risk individuals. Recently, increasing evidences have demonstrated the close link between inflammation and thrombosis, [14, 17–19], which, however, was not well studied in orthopedic trauma field. Thus, the present study was specifically designed to investigate the association of NLR or PLR with preoperative DVT in the setting of a very common fracture type, ankle fracture. We found that elevated NLR and PLR were independently associated with 2.16- and 2.84-fold increased risk of DVT, respectively; also, the combination diagnostic model, including NLR, PLR, and D-dimer, demonstrated to significantly improve the diagnostic performance compared to D-dimer use alone or combined with PLR or NLR (*P* < 0.05).

The ultrahigh sensitivity of D-dimer is helpful in excluding acute DVT or PE, but the limited specificity does not allow diagnostic confirmation, especially in those hospitalized patients [15]. Thus, adding markers to form a practical combination diagnostic model is highly desirable to improve the diagnostic accuracy. During the past decade, more attention has been given to the correlation of inflammatory factors (NLR/PLR) with prognosis (morbidity and mortality) after surgically treated fractures [20–22]. However, few linked them to the thrombosis after fracture. In a retrospective of 1179 tibial plateau fractures, Liu et al. [23] found NLR and PLR were significantly different between patients with and without preoperative DVT in the univariate analysis, but either was no more significant after adjustment for other covariates; instead, the platelet and neutrophil count were identified as independent factors for DVT [23]. In this study, we found both NLR and PLR were independently associated with 2.16-fold and 2.84-fold increased risk of preoperative DVT after ankle fractures, possibly suggesting the fracture locations also contribute a role in risk of DVT.

In previous studies, researchers examined the referral intervals of the PLR and NLR in the population of adult physical examinees. Meng et al. [24] reviewed the data of 24,029 healthy physical examinees in Henan China, and reported the NLR of 1.72 (1.39–2.17) for males and 1.71 (1.35–2.18) for females and PLR of 102 (85–124) for males and 115 (95–140) for females. In another study covering 38,176 adults without any disease and ostensibly healthy in Wuhan China, Fei et al. [25] reported the comparable results, NLR of 1.67 (1.33–2.11) and PLR of 113 (93–137) for males, 1.68 (1.32–2.14) and 124 (102–150) for females, respectively. Similar results were also reported in other studies [26–28]. In contrast, our reported results (NLR, 4.77; PLR, 172) that were 2.8 times and 1.6 times the normal referral value reflected the systemic immune/inflammatory response to fracture trauma (especially the vascular damage around the fracture), which might largely explain the association with DVT identified herein.

The potential pathophysiological mechanisms underlying the association between NLR/PLR are not fully elucidated. In an in vivo, a cross talk was identified between neutrophils, platelets and monocytes, and neutrophil provided the initiating stimuli for formation of thrombus and platelets contributed to the propagation and progression of DVT [29]. In addition, the neutrophils get entrapped in the growing thrombus, enabling recruitment of other cells active in the coagulation cascade via the release of neutrophil extracellular traps (NETs), especially when blood flow is minimal in venous valves [30]. In other animal studies, NK cell-dependent IFN-γ production demonstrated to play a crucial role in formation of NETs by neutrophils for thrombus development [31].

The identified association of PLR/NLR with DVT and the established combination diagnostic model can be used as auxiliary screening tools in management of ankle fractures. For example, they can be used to identify those who carry higher risk of preoperative DVT, and thus prompt and targeted preventive measured can be administered before DUS is arranged, resembling the “empirical prevention.” Because, in most large hospitals, DUS examination is not readily accessible. On the other hand, for patients at low-risk of DVT, e.g., with NLR, NLR and D-dimer level less than the cut-off value, routine chemoprophylaxis or DUS screening is not necessarily prescribed.

This was the first study to specifically investigate the association of NLR/PLR with the preoperative DVT in ankle fracture, and the strengths included strict screening criteria, multiple variables for adjustment and establishment of a combination diagnostic model. Several limitations should also be noted. First, the retrospective design had the inherent limitation in data collection, especially in terms of self-reported comorbidities, body mass and height, lifestyles. However, the primary variables (neutrophil, lymphocyte, platelet count) were hardly affected. Second, although we adjusted for the time from injury to DUS examination, the dynamic change over time for NLR/PLR was not investigated, and should be a future research direction. Third, fracture severity, surrounding soft-tissue damage, degree of vascular injury and immobility of affected limb almost certainly impact the risk of DVT, but relevant data were unavailable or could not be measured. Fourth, the single-center study in a tertiary university-affiliated hospital might have affected the results, because patients transferred were more likely medically unstable or had a complex fracture type. Thus, the generalizability may be less to other settings.

In conclusion, preoperative DVT incidence was 8.3% after ankle fracture. We identified both NLR and PLR as independent risk factors associated with preoperative DVT, with OR of 2.16 and 2.84, respectively. The combination diagnostic model, including PLR, NLR with D-dimer, demonstrated better diagnostic performance than use of D-dimer alone, and could be considered as an auxiliary tool for identifying high-risk patients for subsequent DUS examination. Future studies are warranted for elucidating the dynamic change of PLR/NLR over time after injury and the possible underlying mechanism.

## Data Availability

All the data will be available upon motivated request to the corresponding author of the present paper.

## Abbreviations

NLR: Neutrophil to lymphocyte ratio
PLR: Platelet to lymphocyte ratio
DVT: Deep venous thrombosis
PE: Pulmonary embolism
VTE: Venous thrombus embolism
DUS: Duplex ultrasound
ASA: American Society of Anesthesiologists
BMI: Body mass index
SD: Standard deviation
IQR: Interquartile range
CI: Confidence interval
ROC: Receiver operator characteristic
AUC: Area under the curve
OR: Odd ratio
H–L: Hosmer–Lemeshow
